# Development of a research-based classification of approaches to paediatric palliative medicine service provision within children’s and young adults’ hospices: A mixed methods study

**DOI:** 10.1177/02692163221082423

**Published:** 2022-03-14

**Authors:** Jo Frost, Jane Hunt, Jaqui Hewitt-Taylor, Susie Lapwood

**Affiliations:** 1 Bournemouth University/Children’s Hospice South West, UK; 2 Children’s Palliative Care, University Hospitals Dorset NHS Trust/Hospice Doctor Julia’s House Hospice; 3 Bournemouth University, Dorset, UK; 4 Helen House, Oxford, UK

**Keywords:** Hospices, child, young adult, palliative medicine, palliative care

## Abstract

**Background:**

Globally, pioneers in children’s palliative care influenced this speciality’s development through individual initiatives leading to diverse models of care. Children’s and young adults’ hospices have now been established around the world. However, service provision varies widely leading to inequities both within countries and internationally.

**Aim:**

To describe and classify existing approaches to paediatric palliative medicine in children’s and young adults’ hospices across the UK.

**Design:**

A mixed methods study conducted by telephone interview.

**Setting/participants:**

Thirty-one leaders of children’s hospice care, representing 28 services, 66% of UK children’s and young adults’ hospice organisations.

**Results:**

A geographic-specialist classification was developed through integration of findings, enabling hospices to be classified as *Regional specialist, Regional non-specialist, Local specialist and Local non-specialist.* Both qualitative and quantitative data demonstrated diversity and inequity in paediatric palliative medicine provision. Of 159 doctors (63.5% of whom were general practitioners) working in participating hospices only 27.5% had specialist training in paediatric palliative medicine. The majority of participating hospices (67.9%) did not have involvement from a paediatric palliative medicine consultant.

**Conclusions:**

Internationally, the integration of specialist children’s palliative care teams with existing services is a current challenge. Despite differing approaches to children’s palliative care world-wide, models of care which facilitate integration of specialist children’s palliative care could benefit a range of countries and contexts. The geographic-specialist classification could be used to inform recommendations for a networked approach to paediatric palliative medicine within children’s and young adults’ hospices to promote equity for children with life-limiting and life-threatening conditions.

**What is already known?**

- European and UK standards define specialist children’s palliative care services as those supported by a consultant in paediatric palliative medicine.
- Children’s and young adults’ hospices have developed globally in an ad hoc manner and approaches to paediatric palliative medicine service provision vary widely.
- There are currently no standard international recommendations for the provision of paediatric palliative medicine within children’s and young adults’ hospices.

**What this paper adds?**

- A foundational evidence base of the position of paediatric palliative medicine within children’s and young adults’ hospices at the point of data collection, demonstrating a lack of specialist paediatric palliative medicine involvement and a predominance of general practitioners within this specialist area of work.
- Evidence of diversity and inequity of paediatric palliative medicine within children’s and young adults’ hospices in the UK.
- A broader approach to defining specialist paediatric palliative medicine within children’s and young adults’ hospices to include clinical interactions between regional and local services.

**Implications for practice, theory or policy?**

- The geographic-specialist classification of paediatric palliative medicine service provision within children’s and young adults’ hospices could be developed and used to provide a foundation for a networked approach to paediatric palliative medicine provision within children’s and young adults’ hospices.
- Findings highlight the need for general practitioners and paediatricians working in children’s and young adults’ hospices to achieve and maintain education, training, recognition and practice in paediatric palliative medicine.
- The classification could enable children’s and young adults’ hospice organisations to review current paediatric palliative medicine service provision and inform service development.

## Background

Children’s palliative care is an evolving field, delivered by multidisciplinary teams in a range of settings, providing care to children and young adults with life-limiting and life-threatening conditions^1^ and their families. Globally, pioneers in this speciality influenced it’s development through individual initiatives building on the foundations of the adult hospice and palliative care movement.^2,3^ Since the first children’s hospice, Helen House, Oxford, UK opened in 1984, children’s and young adults’ hospices have been established globally.^2,4,5^ However models of care and access to services vary widely within individual countries and internationally.^3,5,6^

A current, international challenge is integrating specialist children’s palliative care teams^3^ (defined as those supported by a consultant in paediatric palliative medicine or comparable sub speciality training^7–9^) with existing services, including children’s and young adults’ hospices.

Paediatric palliative medicine was recognised as a paediatric sub-speciality in the UK in 2009^10^ with comparable specialist training now available in twenty European countries.^5^ Hospice and palliative medicine became a recognised sub-speciality in the USA in 2008.^3^ Doctors trained in paediatric palliative medicine work with neonates, infants, children and young people with life-limiting and life-threatening conditions, and their families across hospital, hospice and community settings, providing holistic care focussed on quality of life.^11^

The prevalence of life-limiting and life-threatening conditions in 0–19-year olds is rising.^12^ This population’s needs are well established^1,13–15^ but increasingly complex. Whilst evidence to support the benefits of specialist children’s palliative care services is limited^16^ they contribute to reduced end of life intensive care admissions, decreased hospital deaths and more likelihood of community-based end of life care.^17,18^ Whether they impact significantly on symptom burden or quality of life is currently unproven.^17^

Approaches to paediatric palliative medicine provision within children’s and young adults’ hospices in the UK vary.^19^ Whilst some hospices can support complex care and have teams trained in paediatric palliative medicine others have minimal medical provision and focus on social aspects of care.^1^ This pattern is mirrored across Europe.^5^

To date, no clear description and classification of paediatric palliative medicine service provision within children’s and young adults’ hospices exists.^20,21^ The UK commission into the Future of Hospice Care^20^ and the response by the children’s palliative care sector^21^ highlighted the need for evidence-based service development. Clarity on current service provision is required to develop services responsive to the increasingly complex needs of children and young people with life-limiting conditions.^6^ Classifying approaches to paediatric palliative medicine service provision within children’s and young adults’ hospices would provide a basis for service review and development.

Research aim: To describe and classify existing approaches to paediatric palliative medicine service provision in children’s and young adults’ hospices across the UK.

## Design

This paper reports one aspect of a mixed methods study^49^ exploring medical service provision within UK children’s and young adults’ hospices. The wider study additionally explored how differing approaches to paediatric palliative medicine service provision impacted on responses to specific clinical scenarios.

Setting: UK children’s and young adults’ hospices.

Definition: In the UK children’s and young adults’ hospices provide support for children with life-limiting and life-threatening conditions and their families. As independent organisations hospices vary in the model, type and level of care provided.

All children’s hospices in the UK provide short break care, end of life care, specialist play and bereavement support. Some provide day care facilities and home-based care services. Some have a specialist medical team and can support complex care and others focus on social care.^1^

## Population

Inclusion criteria: All UK children’s and young adults’ hospice organisations including individual hospice services, in-patient units or community services and groups of hospice services under one overarching organisation.

Exclusion criteria: Purely hospital-based children’s palliative care teams. Although their interactions with children’s and young adults’ hospice services were addressed from participants’ perspectives.

Thirty-eight children’s and young adults’ hospice organisations fulfilled the inclusion criteria.^22^

Inclusion criteria for individual participants: leader of care representing a hospice organisation, either nursing lead or medical lead if there was one in post.

## Sample

A total population sample of a representative leader of care for all UK children’s and young adults’ hospice organisations. This approach to sampling has previously been employed in related clinical areas.^23^

## Recruitment

Leaders of care of all children’s and young adults’ hospice organisations in the UK were identified and sent an invitation email and participant information sheet via the national UK charity Together for Short Lives. Hospice services with a lead doctor (one working for a children’s hospice service with responsibility for leading medical care) were identified through the Association of Paediatric Palliative Medicine and an invitation email and participant information sheet sent directly to the lead doctor. A hospice organisation might therefore receive two invitations, one to the nursing lead and one to the medical lead.

Informed consent was obtained by response to the email invitation. Verbal confirmation of this consent was obtained before each interview began.

## Data collection methods

Both qualitative and quantitative data were collected through telephone interviews. Numerical questions related to medical service provision and case-load whilst qualitative data were gathered from open-ended questions and case scenarios, an established approach in palliative care service development^24^ which facilitated data triangulation.

The key domains discussed (Box 1) were derived from a previous pilot survey.^25^ The interview tool was developed by: defining what would be measured, selecting scale(s) and generating interview items.^26^ To increase reliability,^27^ detailed questions were written, the choices of answers communicated consistently, key definitions provided and the schedule pre-tested. The drafted schedule was reviewed by experts in the field as a measure of content validity.^26^ An assessment of convergent or discriminant validity^26^ was impossible as no related measures existed (See supplemental material for full interview schedule).

**Box 1.** Domains for inclusion.

Domains relating to medical service provision within children’s and young adults’ hospices 1. Participant demographics 2. Hospice demographics, referrals and caseload 3. Care needs of caseload of children and young adults 4. Approach to medical service provision 5. Doctors working in hospices 6. Nurse Consultant posts 7. 24/7 on call arrangements 8. Consultants in paediatric palliative medicine 9. Outreach into community and in reach into hospitals 10. Clinical case scenarios

Each participant was assigned a unique number and each hospice service a unique letter to maintain anonymity and reduce researcher bias. Data were stored electronically.

## Data analysis

**Figure 1.**
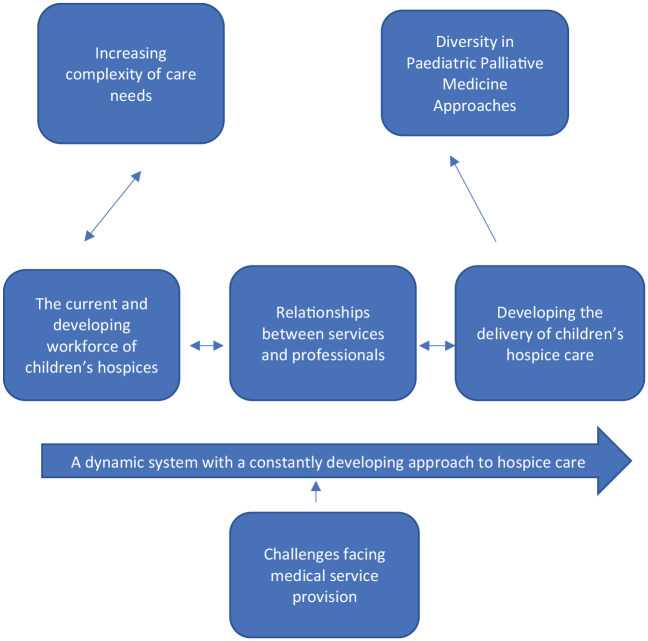
Thematic map derived from qualitative analysis.

Quantitative data were analysed using raw scores and percentages to provide descriptive statistics in relation to medical service provision and caseload. Qualitative data were analysed using thematic analysis,^28,29^ data were coded^30^ reviewed, themes determined and relationships between themes identified to create a thematic map (Figure 1) (See supplemental material for coding frameworks and sub-themes).

### Integration of findings: Development of the geographic-specialist classification

Quantitative and qualitative data were integrated: quantitative data providing information regarding qualifications and levels of provision and qualitative data illustrating the complexity and overlaps between classifications (see Table 1). Commonly used terms were adopted to make the classification meaningful within the speciality.

**Table 1.** Data contributing to a definition of a specialist children’s and young adults’ hospice.

Aspect of the definition of specialist or non-specialist children’s and young adults’ hospice service	Data type	Data source
The presence of a consultant in paediatric palliative medicine	Quantitative	Number of consultants in paediatric palliative medicine
The overall hours of medical presence	Quantitative	Total doctor hours per week plus on call
The level of specialism* in paediatric palliative medicine of doctors	Quantitative	Number of doctors trained to level three and level four in paediatric palliative medicine
The level of specialism* in paediatric palliative medicine of doctors	Qualitative	Within the main themes *Developing the delivery of children’s hospice care* and *Challenges facing medical service provision*
Ability to access paediatric palliative medicine advice 24/7	Quantitative	Data on access to specialist paediatric palliative medicine advice at level three and level four
Ability to access paediatric palliative medicine advice 24/7	Qualitative	Within the main theme *Diversity in paediatric palliative medicine approaches* sub-themes *Diversity in approaches in 24/7 advice* and *The specialist question*
Interaction with NHS services	Quantitative	Numerical data on relationships of hospice services to tertiary children’s hospitals, district general hospitals and neonatal units
The abilities of regional and local services to act as a resource for paediatric palliative medicine advice and review	Qualitative	Within the main theme *Relationships between services and professionals* the subtheme *In reach from hospice to hospital*

Approaches to paediatric palliative medicine service provision were defined at the intersection of geographic considerations and based on the level of specialist paediatric palliative medicine service provision. The geographical definition drew on qualitative data from a main theme *Diversity in paediatric palliative medicine approaches* and quantitative data regarding referral area, caseload size, in-patient hospice unit numbers, outreach service numbers and relationships to NHS hospitals. These were synthesised to define ‘regional’ and ‘local’ hospices.

Defining hospices as ‘specialist’ or ‘non-specialist’ utilised qualitative data from subthemes *The specialist question* and *Diversity in approaches to 24/7 medical advice* and the main themes *Developing the delivery of children’s hospice care* and *Challenges facing medical service provision* alongside quantitative data regarding: doctors’ weekly hours, doctors’ level of specialist training and education, access to 24 h a day 7 days a week paediatric palliative medicine advice. These data were synthesised for all hospices in the study and parameters for ‘specialist’ and ‘non-specialist’ provision defined.

The definition developed considered the following aspects supported by quantitative and qualitative data (Table 1).

This definition of specialist paediatric palliative medicine within children’s and young adults’ hospices encompasses the previous narrow definition of a specialist children’s palliative care service^7,8^ but the geographic-specialist classification additionally incorporates how regional and local services interact in a networked approach.^31^ For a local hospice to be defined as ‘specialist’ in paediatric palliative medicine both the level of education and training of doctors working there and the interaction with a regional specialist hospice with a consultant in paediatric palliative medicine are considered.

### Ethical approvals and issues

Bournemouth University Research Ethics Committee approved the study (12/09/14, Ref 4189). The study was also reviewed by all participating children’s and young adults’ hospice organisations’ individual research or governance groups.

## Results

Thirty-one leaders of children’s hospice care participated in the study. In three cases this included both medical and nursing leads. The 31 interviews relate to 28 children’s and young adults’ hospice services (Table 2), representing 25 children’s and young adults’ hospice organisations, a response rate of 66% (2015).^22^ Whilst data were collected in 2015 no classification of this type has been developed since. Although children’s hospice organisations have increased in number and service configurations altered, the issues of diversity in provision and need for integration of paediatric palliative medicine remain current.^3,6^

**Table 2.** Description of participating hospice services.

Configuration of hospice service	Number of hospice services (*n* = 28)	Number of hospice services in this category with arrangement for formal medical cover of any description (%)
Stand-alone community hospice service	4	2 (50%)
Hospice with inpatient unit for children	3	3 (100%)
Hospice with inpatient unit for children and young adults	2	2 (100%)
Joint inpatient and community outreach for children	6	6 (100%)
Joint inpatient and community outreach for children and young adults	9	9 (100%)
Children’s hospice as part of an adult hospice organisation	4	4 (100%)

Table 3(a) to (e) summarises key descriptive data relating to levels and specialism of medical service provision.

**Table 3.** (a) Number of doctors working at participating hospice service.

Number of doctors (as a range)	No. (%)
0	3 (10.7)
1–4	10 (35.7)
5–9	9 (32.2)
10 and over (max 13)	6 (21.5)
Total	28 (100)

**Table:** (b) Doctors hours in direct patient care per week at participating hospice service.

Doctors’ hours in direct patient care per week (as a range)	No. (%)
0	4 (14.3)
1–30	13 (46.4)
30–60	6 (21.5)
60–90	2 (7.1)
90–120	2 (7.1)
Answered: Don’t know	1 (3.6)
Total	28 (100)

**Table:** (c) Background specialty of doctors at participating hospices.

Background specialty of doctors at participating hospices	No. (%)
General practice	82 (51.6)
General practitioner with special interest in paediatric medicine	18 (11.3)
Consultant in adult palliative medicine	10 (6.3)
Paediatric palliative medicine consultant	10 (6.3)
Paediatric intensive care	5 (3.15)
Paediatric oncology	5 (3.15)
Community paediatrician	8 (5)
Paediatrician with special interest in paediatric palliative medicine	7 (4.4)
Specialist registrar paediatrics	4 (2.5)
General paediatrician	4 (2.5)
Other or not specified	6 (3.8)
Total	159 (100)

**Table:** (d) Level of specialism in paediatric palliative medicine of doctors at participating hospices.

Level of specialism in paediatric palliative medicine of doctors at participating hospices*	No. (%)
Level three	32 (20.1)
Level four	12 (7.5)
None	115 (72.4)
Total	159 (100)

**Table:** (e) Type of 24/7 medical advice at participating hospices.

Type of 24/7 medical advice at participating hospices	No. (%)
Specialist paediatric palliative medicine (Level 3 or Level 4*) 24/7	14 (50)
Generic medical advice with augmentation for end-of-life care	6 (21.4)
Generic medical advice only	5 (17.8)
No formal 24/7 medical advice	3 (10.8)
Total	28 (100)

* At the time of data collection the Association of Paediatric Palliative Medicine and Royal College of Paediatrics and Child Health defined levels of specialist training from one to four in paediatric palliative medicine (combined curriculum).^32^ with consultants trained in paediatric palliative medicine according to the Royal College of Paediatrics and Child Health curriculum^33^ equivalent to level four and hospice doctors or paediatricians with specific additional training in paediatric palliative medicine at level three.

Qualitative data enhanced understanding of the elements and complexity of provision. For example, participant 20/Q described ‘*the combination of management in one child, for example a child with a stoma, a tracheostomy and central line*’. This was combined with changing service expectations, as stated by participant 28/Y: ‘*in the past these children would never have come out of hospital*’. and linked to an increasing need for specialist training and skills: with participant 5/E explaining: ‘*we have difficulty maintaining competency and keeping up to date. . . barriers to upskilling the team have been the catch 22 of no ventilated patients, therefore we are not able to develop skills, therefore there are no ventilated patients*’.

Qualitative and quantitative data similarly facilitated the development of the classification by demonstrating key characteristics of hospices. For example, Participant 25/V explained: ‘*the children’s hospice is described as a local hospice as opposed to a regional hospice*’. Additionally, participant 22/S related the hospice’s workload to geographical factors: ‘*the hospice is set in a rural and large area in comparison to a hospice near a tertiary children’s hospital. The workload is variable, and the skills needed are variable*’. This, alongside quantitative data regarding the hospice’s medical service provision and relationship to tertiary and district general hospitals contributed to the concept of regional versus local services.

Participants also used the term ‘specialist’ regarding hospice services, with Participant 27/X explaining: ‘*Three years ago I would argue that we weren’t even delivering palliative care let alone specialist palliative care. Previously we were a respite unit. . . we made a decision not to be a respite unit for complex disability and we transitioned from respite to palliative care. This came out of strategy. We had to turn down commissioned work and transitioned children. We had to upskill staff. This was a painful process but the right process*’. This concept of specialist versus non-specialist was developed in combination with numerical data regarding the level and specialism of medical service provision to define specialist versus non specialist hospices.

### Geographic-specialist classification

The main outcome of this stage of the study was the development of a classification of approaches to paediatric palliative medicine service provision within children’s and young adults’ hospices across the UK. The resulting geographic-specialist classification represented the majority (24) of participating hospices (28).

This classification comprises four groups: (1) *Regional specialist, (2) Regional non-specialist, (3) Local specialist and (4) Local non-specialist* (Table 4). As it is derived from the real and diverse world of children’s hospice care, the words ‘usually’, ‘often’ and ‘may’ are employed, indicating a need for flexibility in the definitions.

**Table 4.** Summary of characteristics of geographic-specialist classification.

	Regional specialist	Regional non-specialist	Local specialist	Local non-specialist
Number of participating hospices in classification	6	3	7	8
Referral area	One region* (or majority of region for larger regions)	One region (or majority of region for larger regions)	Geographical section of a region	Geographical section of a region
Inpatient units	Usually >1	Usually >1	Usually 1	1 or none
Outreach services**	At least one	Variable	1 or none	1 or none
Caseload of children and young people	>250	>250	Usually <250 or may be higher	Usually <250
Number of doctor hours	High, usually >40 per week plus on call	Fewer doctor hours, may be less than 40 h a week plus on call	High, may be >40 per week plus on call	Significantly fewer doctor hours <40 h per week plus on call
Level of specialist training of doctors	One or more level 4 or consultants in paediatric palliative medicine. Or >3 level 3, usually with a formal or informal link to a level 4	No level 4 <3 level 3	At least one level 3 or more. May be linked to a level 4	No level 3 or level 4 link
24/7 paediatric palliative medicine advice	System for 24/7 access to paediatric palliative medicine advice including level 4 advice or robust system for level 3 advice	No robust system for 24/7 access to paediatric palliative medicine advice. May have augmented cover for end of life care.	System for access to 24/7 paediatric palliative medicine advice at level 3. May have link to level 4 when needed	No system for 24/7 access to paediatric palliative medicine advice
Relationship to NHS hospitals	Close relationship with tertiary children’s hospital offers medical in-reach to a number of district general hospitals and neonatal units.	Intermittent or limited relationship with tertiary hospital, district general hospitals or neonatal units. In particular no medical in-reach.	Close relationship to local district general hospital and neonatal unit including medical in-reach.	Limited relationship with local district general hospital or neonatal unit. In particular no medical in-reach.

* A region is the highest tier of sub-national division in England.

** Outreach service defined as: community-based hospice services with an element of medical or nursing outreach rather than purely bereavement support, play support or short breaks that are non-nursing.

#### Additional approaches

In addition to the geographic-specialist classification, three ‘outlier’ approaches to paediatric palliative medicine service provision were described: (i) *One-person pioneer*, (ii) *Nurse led 24/7 paediatric palliative medicine* and (iii) *Rural hospice*. These represent important alternative approaches demonstrated in 4 of the 28 participating hospice services (see Table 5).

**Table 5.** Summary of characteristics of alternative classifications.

	One-person pioneer	Nurse led 24/7 paediatric palliative medicine	Rural
Referral area	Variable may be a region or part of a region	Usually a geographical part of a region	A geographical section of a region
Inpatient units	Usually 1	1 or none	Usually 1
Outreach services	1 or none	1 or none	1 or none
Case load of children and young people	Usually high May be >250	Usually lower <250	<250
Number of doctor hours	Usually fewer hours as only one doctor <40 h per week plus on call	Low number of doctor hours	Fewer doctor hours, usually significantly less than 40 h a week
Level of specialist training of doctors	Usually level 4 based	Level 3 or no level 3 or 4. Doctors as a resource rather than lead	Variable, may have one level 3 doctor
24/7 Paediatric palliative medicine advice	One person PPM 24/7 may be supported by 24/7 nursing rota	24/7 nurse consultants and nurse prescribers	Usually no robust system for 24/7 access to paediatric palliative medicine advice. May have augmented system for end of life care or informal Level 4 link
Relationship to hospitals	Close relationship to tertiary hospitals and district general hospitals	In reach in local hospitals led by nurses	No nearby hospital either District general or tertiary

### Discussion

The global children’s hospice movement has been led by individual initiative and independent pioneering,^2,34^ creating diverse approaches to service provision and models of care.^1,2,5^ This study highlights that diversity in relation to paediatric palliative medicine service provision within UK children’s and young adults’ hospices and proposes a classification to clarify the integration of specialist children’s palliative care within these hospices.

The European Association of Palliative Care (EAPC) atlas^5^ identifies three main types of children’s palliative care services; inpatient hospices, hospital-based children’s palliative care programmes and home care programmes for children’s palliative care. Hospice services demonstrate diverse approaches to care provision.^5^ Some countries, including the UK, report children’s palliative care as integrated throughout health care systems.^5^ However, many countries in the WHO Europe Region have no designated palliative care for children.

In the USA most paediatric hospitals have paediatric palliative care teams.^35^ In contrast, the pioneering children’s hospice services in the USA^2^ are the exception.^9,36^ Nonetheless, the American Academy of Paediatrics are committed to hospitals providing care for children with life-limiting conditions having dedicated interdisciplinary speciality paediatric palliative care and paediatric hospice care teams.^9^

Historically, evidence concerning global children’s palliative care provision has been limited. The first international overview of children’s palliative care^37^ identified six levels of development. However, as the UK is amongst the seven countries placed in the highest category of provision^37^ the lack of integration of paediatric palliative medicine and limited specialist training for doctors working in children and young adults’ hospices identified in this study is internationally relevant.

Since the development of training in children’s palliative care^2,38^ significant worldwide progress has occurred, including sub-specialist training in paediatric palliative medicine.^5,9^ This includes European initiatives^5^ and a curriculum in paediatric palliative care developed in the USA with potential for use world-wide.^39^ Nonetheless, despite recognised specialist medical education and training curricula in the UK^11,32^ the majority of doctors working in children’s and young adults’ hospices at the time of data collection had no specialist training in paediatric palliative medicine.

Original UK guidelines for good practice in children’s and young adults’ hospices^40,41^ have been superseded by detailed national and international standards regarding children’s palliative care^1,7,9^ and end of life care^42^ in all settings. However, there are currently no agreed standards or expectations specific to children’s and young adults’ hospices. Therefore, as evidenced by this study, diverse approaches to service delivery have developed.

That meeting the needs of children and young adults with life-limiting and life-threatening conditions requires a comprehensive, multi-disciplinary team, working in a coordinated and integrated manner is well established.^1,7,9,43^ Although no one correct approach to children’s palliative care provision exists^44^ the aspiration for equitable service provision is affirmed internationally^45,46^ alongside increasing evidence regarding the benefits of timely involvement of specialist children’s palliative care teams.^17^ Achieving consistent integration of specialist children’s palliative care within health care systems is recognised as a key future challenge.^3^ (Sisk, Feudtner and Bluebond-Langner, 2020) Despite vastly differing access and approaches to children’s palliative care world-wide, the geographic-specialist classification described here could be developed and used as a foundation to achieve this goal.

Strengths/limitations of the study: The interviewer being known to be a paediatrician working in children’s palliative care may have affected responses. Additionally, the study represents a ‘snapshot’ of service provision in 2015. However, the issues of diversity in provision and need for integration of paediatric palliative medicine and children’s hospice care remain topical.^3,6^

## Conclusions

This study identifies the need for equitable access to specialists in paediatric palliative medicine whilst enabling general practitioners and paediatricians working in children’s and young adults’ hospices to achieve and maintain training, recognition and practice in paediatric palliative medicine. Whilst this finding was specific to the UK, the need for integration of specialist children’s palliative care within health care provision is an international issue.

The geographic-specialist classification could be developed and applied to facilitate improved integration of children’s hospice care and paediatric palliative medicine. For example, classifying and linking local and regional services could improve access to specialist paediatric palliative medicine and provide training opportunities for doctors working in local services.

In England the commissioning and delivery of specialist children’s palliative care services within managed clinical networks is recommended.^31,47^ These aim to provide equitable services and improved care by enhancing collaboration between primary, secondary and tertiary professionals and their organisations.^31^ Children’s and young adults’ hospices are proposed as best placed to lead this development.^47,48^ Therefore, the classification proposed could be developed and applied within a managed clinical network within England.

The classification could be used to compare outcomes for specialist and non-specialist children’s and young adults’ hospice services. In addition, further research into alternative approaches to paediatric palliative medicine service provision, particularly the role of nurse led services, is needed.

## Supplemental Material

sj-pdf-1-pmj-10.1177_02692163221082423 – Supplemental material for Development of a research-based classification of approaches to paediatric palliative medicine service provision within children’s and young adults’ hospices: A mixed methods study Click here for additional data file. Supplemental material, sj-pdf-1-pmj-10.1177_02692163221082423 for Development of a research-based classification of approaches to paediatric palliative medicine service provision within children’s and young adults’ hospices: A mixed methods study by Jo Frost, Jane Hunt, Jaqui Hewitt-Taylor and Susie Lapwood in Palliative Medicine

sj-pdf-2-pmj-10.1177_02692163221082423 – Supplemental material for Development of a research-based classification of approaches to paediatric palliative medicine service provision within children’s and young adults’ hospices: A mixed methods study Click here for additional data file. Supplemental material, sj-pdf-2-pmj-10.1177_02692163221082423 for Development of a research-based classification of approaches to paediatric palliative medicine service provision within children’s and young adults’ hospices: A mixed methods study by Jo Frost, Jane Hunt, Jaqui Hewitt-Taylor and Susie Lapwood in Palliative Medicine
