# Supplementary material for: Development of a research-based classification of approaches to paediatric palliative medicine service provision within children’s and young adults’ hospices: A mixed methods study
Source: Palliat Med. 2022 Mar 14;36(5):855–65. doi: 10.1177/02692163221082423 (PMC9087311; doi:10.1177/02692163221082423)
Supplement: sj-pdf-1-pmj-10.1177_02692163221082423 – Supplemental material for Development of a research-based classification of approaches to paediatric palliative medicine service provision within children’s and young adults’ hospices: A mixed methods study [file sj-pdf-1-pmj-10.1177_02692163221082423.pdf]

Diagrams of main themes with sub-themes and coding frameworks

(For overall thematic map see Figure one in main manuscript)

Diagram for main theme: Increasing complexity of care needs

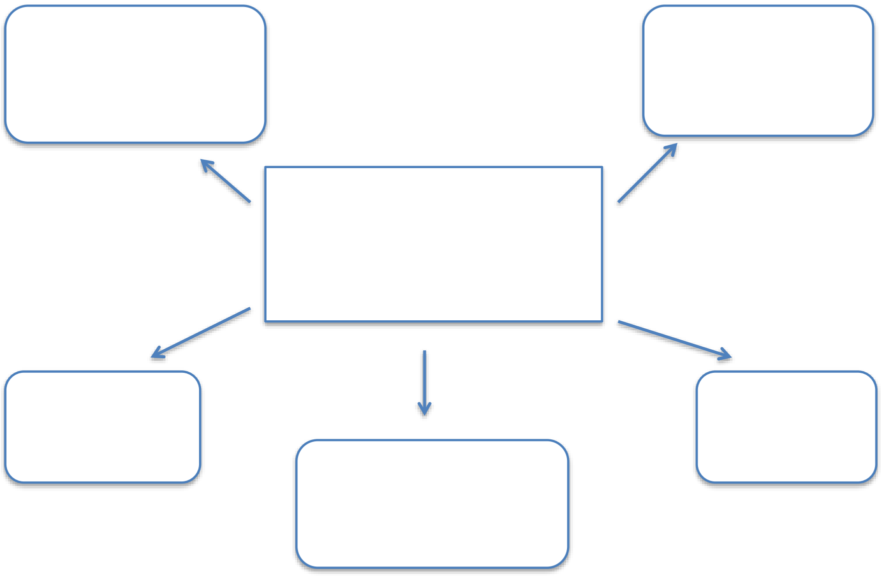

Coding framework for the main theme: *Increasing complexity of care needs*

Subtheme	Code name	Description	Example
Increasing complexity of conditions	The whole picture	The whole picture will refer to any reference to a combination of multiple needs within one child	"*It is not one individual care need but the combination that leads to complexity…not the individual pieces but the whole picture"*
Increasing complexity of conditions	Complex symptoms	Complex symptoms will refer to any reference to the symptoms experienced by the child increasing in complexity	'*Unstable symptoms, managing symptoms and juggling medications'*

Increasing complexity of conditions	Challenging behaviours	To refer to any reference to challenging behaviour contributing to increasing complexity of care	*12/J "Neurodevelopmental conditions with behavioural needs such as autism spectrum disorder and challenging behaviour"*
Increasing complexity of conditions	Epilepsy care	To refer to any reference to the management of epilepsy increasing the complexity of care	19/P "*Seizure management is challenging, for example seizures leading to ITU admission"*
Increasing complexity of interventions	Developments in technology	To refer to any reference to a technological intervention increasing the complexity of care including respiratory, ostomies, haemodialysis, catheters	13/K "*Stoma care [adds to the complexity of care], often multiple stoma care, for example; urostomy, ileal conduit, nephrostomy. Increasingly children have complex combinations of these."*
Increasing complexity of interventions	Medications	To refer to any reference to the use of medications as an intervention increasing the complexity of care: including new medication, new modes of delivery of medication and rotation of opiates.	"*admission for opiate rotation including on to methadone and the use of ketamine. We arrange admission [to the hospice] to initiate… we also arrange admissions to establish medications"*
Changing parameters of care	Changing place of care	To refer to any reference to a change in the place a child is cared for adding to the complex care at a hospice	28/Y "*In the past these children would never have come out of hospital"*
Changing parameters of care	Changing age profile	To refer to any reference to a change in the age of children receiving hospice care	21/R *" There is a changing dynamic, previously we provided respite and play therapy. [The hospice] started with 120 children and now 80 -90% are less than 3yrs old*

			therefore there is a different profile, including antenatal referrals.'
Changing parameters of care	Changing goals of care	To refer to any reference to a change in the expected outcome of an illness for child	9/H "*Initially this child [at hospice requiring haemodialysis] was for palliation but is now on the transplant list."*
Changing parameters of care	Changing referral patterns	To refer to any reference to a change in a the way in which referrals to hospices are influencing the complexity of care	5/E *"We have been working with oncology services around referrals. Previously we only accepted when a child was terminally ill. We have changed our criteria to emphasise the life threatened group of children with cancer."*
Ethical issues and facilitated decision making	Ethical issues and facilitated decision making	To refer to any reference to ethics or involvement in ethical decisions such as withdrawal of treatment	7/G *" Other admissions occur for withdrawal of other types of treatment for high dependency unit, oncology patients."*
Hospice related factors	A can do approach	To refer to any reference to the hospice adapting to meet new needs	21/R "*We would rise to any challenge".*
Hospice related factors	Distance to hospital/ transport issues	To refer to any reference to the distance to hospital or issues with transport impacting on the complexity of care	22/Q ""*For children with epilepsy and major seizures or status. The care provided is complicated by the distance to hospital. There has been consideration of giving one patient a port for IV access for this reason"*

Diagram for main theme: ***Diversity in approaches to PPM service provision***

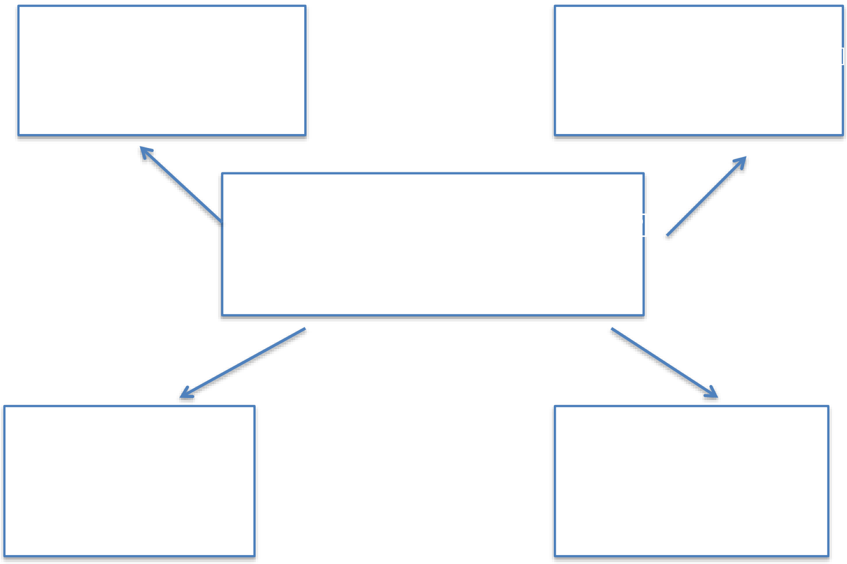

Coding framework for main theme: ***Diversity in approaches to PPM service provision***

Subtheme	Code name	Description	Example
Defining the overall hospice classification	Geographical models (includes regional, local and rural)	To refer to any reference to geography as a defining factor in the hospice classification	25/V *" the children's hospice is described as a local children's hospice as opposed to a regional hospice."*
Defining the overall hospice classification	Linked children's and adults hospice	To refer to any reference to a children's hospice linked to an adult hospice	27/X *"A five bedded inpatient hospice attached to an adult hospice. From the care point of view the adult and children's hospices support each other as one hospice that takes all ages. The hospices were built independently in he same grounds and ten years ago came together."*
Defining the overall hospice classification	Profession led models (includes nurse led, one person pioneer	To refer to any reference to the profession of the leader of care as	*3/C "nurse led with a link to local GPs."*

	and medical teams)	defining the hospice model	
Diversity in approaches to 24/7 PPM advice	Service Level agreement models	To refer to any reference to a method of providing 24/7 medical advice	20/Q "We have an *SLA with a GP Coop for medical advice on general issues level one and two"*
Diversity in approaches to 24/7 PPM advice	In house models	To refer to any reference to the provision of 24/7 medical advice by the regular hospice doctors	13/K *"A team of doctors doing daily sessions and covering on call. 2- 4 hour sessions on weekdays covered by GPs and paediatricians. Regular sessions in weekends"*
Diversity in approaches to 24/7 PPM advice	Augmented for end of life care models	To refer to any reference to an increase in 24/7 medical cover for end of life care	24/U *"A 24/7 on call rota is run by the hospice, this is set up responsively for end of life care with doctors from the hospice"*
Diversity in approaches to 24/7 PPM advice	Multi-layer models	To refer to any reference to a combination of different levels of 24/7 medical advice for one hospice service	27/X *"A 24/7 on call rota of doctors is run by the hospice and we have an SLA with a tertiary children's hospital. This is a two tier system: a rota by regular doctors and back up by [Tertiary children's hospital] 24/7."*
Diversity in approaches to 24/7 PPM advice	Regional models	To refer to any reference to a hospice providing medical advice (usually PPM advice) for a region	9/H *"A managed clinical network run a 24/7 rota. There is a 24/7 nurse on call rota of nurse specialists and a doctor on call rota of Level three PPM doctors."*
Diversity in approaches to 24/7 PPM advice	No 24/7 medical advice models	To refer to any reference to a hospice with no formal system for access to 24/7 medical advice	*26/W "we have no access to 24/7 medical advice. There is a nurse led 24/7 on call rota of senior nurses all prescribers".*

The specialist question	Concerns regarding a specialist model	To refer to any reference to a concern in relation to specialist PPM	1/A . *" [I am] not convinced that having a PPM consultant is the only way to deliver the expertise needed. Local expertise also needs to be utilised. I'm concerned about the palliative care funding pilot and whether only Level four services will have access to specialist funding."*
The specialist question	Who delivers specialist PPM care?	To refer to any reference to a professionals as being specialist in PPM and therefore involved in the delivery of PPM	1/A *"The lead for medical service provision by the hospice service is a paediatrician with a special interest in PPM, level three trained, contracted by the hospice."*
Diversity in approaches to outreach	Type of outreach	Refers to any reference to the practical focus of the outreach ie nursing, respite, play therapy, sibling support	26/W *"The complexity of the child depends on who is sent home. Visits can be play support, nursing outreach or sibling support. We can also use family volunteer support workers."*
Diversity in approaches to outreach	Configuration of outreach services	Refers to any reference to the way in which the outreach service operates	26/W "*A Nurse and therapist lead a team that is integrated and works across inpatient and community settings. 70 % of our work is in the community"*

Diagram for main theme: *The current and developing workforce of children's hospices*

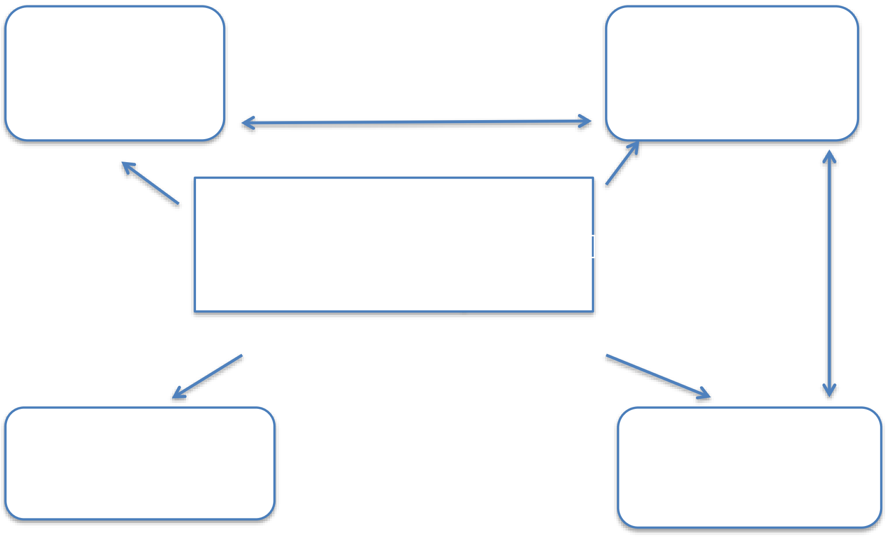

Coding framework for main theme: *The current and developing workforce of children's hospices*

Subtheme	Code name	Description	Example
The foundation of General practice	The foundation of General Practice (NB this code was so significant it became a subtheme)	To refer to any reference to GPs or a general practice playing a part in the medical cover for a hospice	2/B **"***The hospice has a link with a local General Practice, it is a three man GP surgery and one of the GPs works for the hospice and is very dedicated."*
The nursing versus medical role. A fading distinction?	The nursing versus medical role. A fading distinction? This code became a subtheme	To refer to any reference to nurses in medical or specialist PPM roles	7/G "*The nurse Consultant has an honorary contract with hospitals. The specialist PPM review is done with support of the [hospice] medical team."*

The current workforce of children's hospices	Medical teams	To refer to any reference to a team of doctors as part of the current workforce at a hospice	7/G *"The team is a mixture of GPs and Paediatricians. Regular clinical sessions are; Consultant PPM: (6 ½ sessions), Consultant PM (6 sessions), Level 4 doctor with research interest (6 hours), Consultant PPM (6 hours), Oncology Staff Grade (6 hours) Adult PM Consultant (18 hours), GP with special interest (24 hours) and a registrar from PICU. "*
The current workforce of children's hospices	Consultants in PPM and adult PM	To refer to any reference to consultants in PPM or adult PM consultants as part of the current workforce	6/F ***"****The Hospital employs the PPM* consultant *and has an SLA with the hospice. The consultant post had college approval as a PPM post"*
The current workforce of children's hospices	Allied professionals	To refer to any reference to allied professionals as part of the current workforce	14/L"*A part time psychologist works 32 hours a week and gives holistic support. She is a vital part of the team."*
Workforce innovations	Workforce innovations: includes review of medical services, creation of new posts.	To refer to any new development in the hospice workforce	20/Q *"We have a lead nurse in transition who is working with adult services. 18months ago we appointed a lead nurse for neonates who is building relationships with NICU as neonatal services have developed".*

Diagram for main theme: Relationships between services and professionals

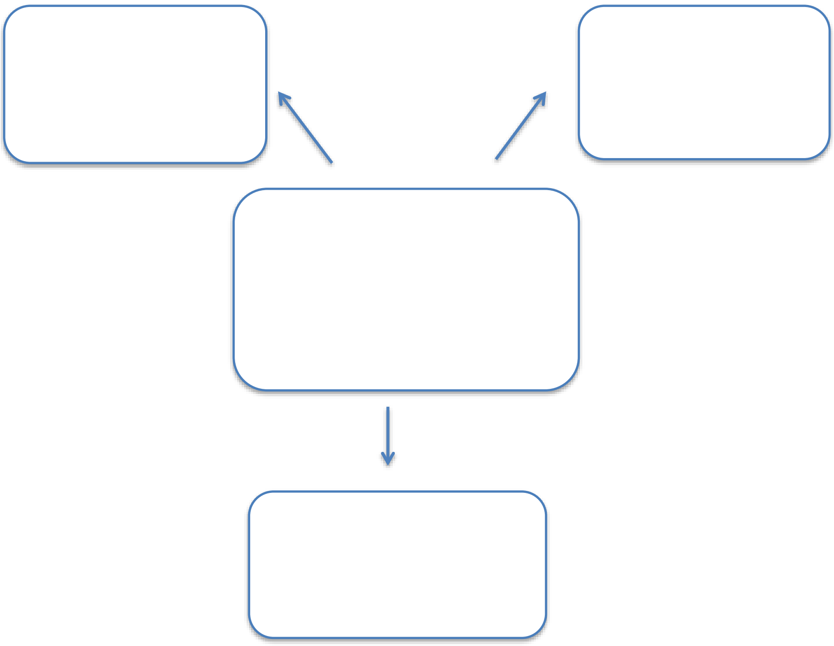

Coding framework for main theme: *Relationships between services*

Subtheme	Code name	Description	Example
Formal arrangements between services	Service level agreements	To refer to any reference to an SLA linking two palliative care services	11/I "There is an *SLA with [Tertiary children's hospital] for OOH telephone support for the children under [Tertiary children's hospital]"*
Formal arrangements between services	Operational pathways	To refer to any reference to a formal pathway to support services working together	10/H "*There is* a *pathway for rapid transfer from PICU and NICU"*
Formal arrangements between services	Communication between services	To refer to any formal strategies for supporting communication between services	26/W *"We are developing a children's multi disciplinary team meeting to discuss all palliative children"*

Relationships between professionals	Professional boundaries and medical leadership	To refer to any reference to systems to ensure professional boundaries and clear medical leadership	9/H "*There is a principle within the team that each child has a lead consultant and it is their patient, we provide palliative care input for them under the lead consultant. This is part of the organisational boundaries and governance. For end of life plans the clinical accountability is with the lead consultant"*
Relationships between professionals	The importance of good working relationships	To refer to any reference to good working relationships supporting care delivered	10/H *"We do not have formal agreements with hospitals but good relationships based on positive outcomes. We've done good stuff together"*
In reach from hospice to hospital	In reach from hospice to hospital	To refer to any reference to the hospice providing a service in an NHS hospital or for an NHS hospital	15/M "*There is in reach into [hospital] and we have three honorary* contracts *but they are not NHS funded"*

Diagram of main theme: Developing the delivery of children's hospice care

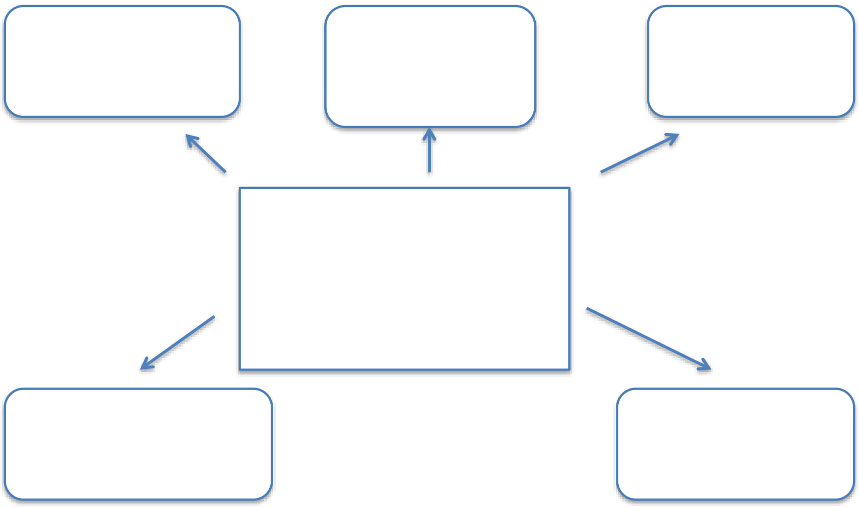

Coding framework for main theme: Developing the delivery of children's hospice care

Subtheme	Code name	Description	Example
Innovations in hospice services	Acceptability of hospice care "A way in"	To refer to any reference to a development in service delivery to improve the acceptability of hospice care	20/Q "From *oncology we previously had last minute end of life referrals. We have developed links with the oncology team. Now most children are referred through the counseling service and sibling support"*
Innovations in hospice services	Clinics	To refer to any reference to a hospice led clinic	23/T "[Tertiary Hospital] *Consultant in PPM runs a clinic with a social worker, a physiotherapist and a specialist nurse, who is a nurse prescriber.* [Tertiary Hospital] Consultants in PPM cover different sides of

			[region] At clinic we see four children in a day."
Innovations in hospice services	Telephone advice	To refer to any reference to a development in telephone advice	*21/K "It is our aspiration to run 24/7 advice with access for the whole of [Region] for medical advice"*
Innovations in hospice services	Allied and holistic care	To refer to any reference to a development in allied or holistic care	*23/T "We are developing a holistic centre for yoga and music"*
Innovations in hospice services	Targeting specific groups	To refer to any reference to a development focused on targeting specific groups of children	7/G "*Populations of children in NICU and Oncology are being targeted."*
Innovations in hospice services	Referral developments	To refer to any development or innovation in the area of referrals	19/P "*We are expanding the catchment area in line with adult service"*
Developments in facilities	Developments in facilities	To refer to any reference to a development of hospice facilities and buildings	1/A: "*A build for an inpatient unit in progress and registered with CQC [Care Quality Commission] for an inpatient unit and community hospice care"*
Developments in outreach services	Developments in outreach services	To refer to any reference to a development in hospice outreach services	7/G "*The outreach service in its current form has only been running for two years. It is actively being developed."*
Developments in education and research	Developments in education and research	To refer to any reference to new initiatives in education or research	15/M "*We are increasingly involved in research by [University] under [lead researcher] aiming to find out the number of children and young people with palliative care needs in [Region] and where are they. The [University] work identified 4000 under*

			25 yr olds"
From strategy to change	From strategy to change	To refer to any reference to strategy as a driver for development	3/C " *We are actively trying to change the current situation where there is no specialist PPM input. This is by the publication of a [region] children's palliative care strategy and appointment of a consultant in PPM"*
From strategy to change	The use of pilots	To refer to any reference to the use of a pilot for service developments	26/W "*We are looking for funding to pilot children's register"*

Diagram of main theme: Challenges facing medical service provision

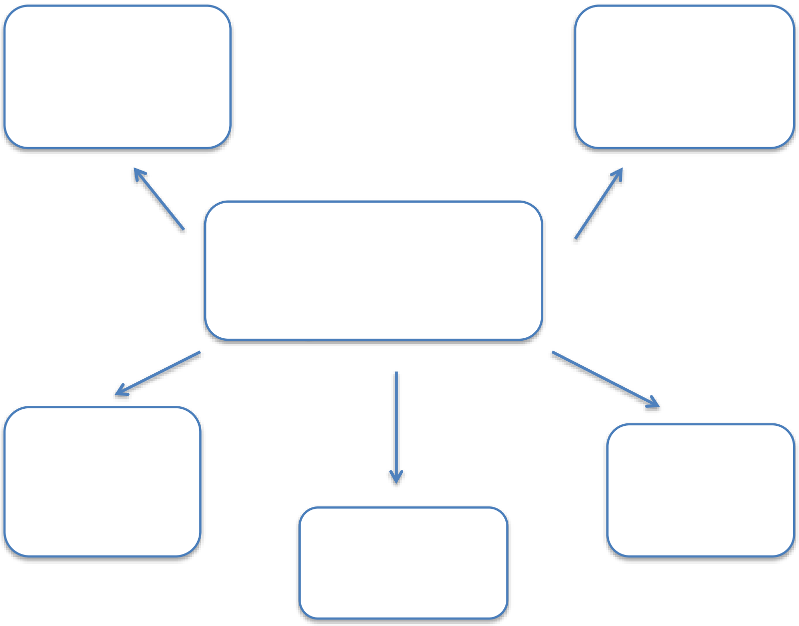

Coding framework for main theme Challenges facing medical service provision within hospices

Subtheme	Code name	Description	Example
Sustainability	Unsustainable 24/7 medical advice	To refer to any reference to a unsustainable provision of 24/7 advice	21/R *“We have 24/7 cover but this is one person therefore not sustainable 24/7”*
Sustainability	Reliance on goodwill	To refer to any reference to goodwill as a factor in hospice doctors work	11/I “*The system relies on the good will of one person.”*
Variable workload	Responsive hours	To refer to any reference to doctors working responsively	21/R “*the doctors regular hours are eight and a half hours but they are responsive to need.”*
Variable workload	Supporting end of life care	To refer to any reference to the variable workload in supporting end of life care	2/B outlined, “the *doctors hours are variable depending on if there is end of life care at the hospice. For end of life care one hour a day is needed* and *therefore seven hours a week. If there is no end of life*

			care they do pop in visits."
Workforce challenges	Maintaining competencies	To refer to any reference to the challenge of maintaining staff competencies	9/H "*It is very difficult to do [haemodialysis] with respect to governance, competencies and difficult to maintain."*
Workforce challenges	Need for increased staffing ratios	To refer to any reference to a need for increased staff ratios	24/U "*Challenging behaviours lead to significant training needed for restraint. And a need for 2 to 1 staff"*
Workforce challenges	Need for peer review	To refer to any reference to needing advice/ support from peers	15/M "*Sometimes need to call on national colleagues for advice*"
Workforce challenges	Recruitment difficulties	To refer to any reference to difficulty in recruitment	2/B **"***It has eight bed inpatient unit but is only running five beds currently in view of staff shortages."*
Workforce challenges	Lack of training posts	To refer to any reference to the challenge of lack of training posts	3/C "*We have no Consultant in PPM in [Region] and no SpR training posts so it is difficult for interested registrars to develop a special interest*."
Medical cover not fit for purpose	Medical cover not fit for purpose	To refer to any reference to problems with the current medical service provision	3/C "*The current medical model does not serve us well… We need someone bringing something to the table, we have to lead the GPs 99% of the time and they are not proactive. We find in terms of the medical model that we can't actively offer as much symptom management as we want to*"
Financial challenges	Financial challenges	To refer to any reference to finances as a challenge in children's hospice care	
