# Supplementary material for: Development of a research-based classification of approaches to paediatric palliative medicine service provision within children’s and young adults’ hospices: A mixed methods study
Source: Palliat Med. 2022 Mar 14;36(5):855–65. doi: 10.1177/02692163221082423 (PMC9087311; doi:10.1177/02692163221082423)
Supplement: sj-pdf-2-pmj-10.1177_02692163221082423 – Supplemental material for Development of a research-based classification of approaches to paediatric palliative medicine service provision within children’s and young adults’ hospices: A mixed methods study [file sj-pdf-2-pmj-10.1177_02692163221082423.pdf]

Interview schedule

(This interview schedule was used as a guide for the interviewer and was not given to the participants).

Participant's details

1. What is your job title?
2. What role do you have within your hospice?
3. What professional qualifications do you have to enable you to undertake your post? (Please include both a) generalist and b) specialist qualifications.
4. How long have you been in your current children's palliative care post (in months)?

Hospice details

5. In which region is your hospice located:

a. Scotland
b. Northeast
c. Northwest
d. Yorkshire and Humber
e. East Midlands
f. West Midlands
g. East of England
h. London
i. Southwest
j. Southeast
k. South Central
l. Wales
m. Northern Ireland

6. What is the geographical area from which your hospice service takes referrals?

a. Regional catchment area

Yes / No

6ai. If yes how many regions does your hospice service accept referrals from

b. A Clinical commissioning group catchment area (Or Health board area for Wales and Scotland or Trust area for Northern Ireland)

Yes/ No

6bi. If yes how many CCG’s/ health board areas or Trust areas does your hospice take referrals from?

7. What is the age range of children and young people for whom your hospice service accepts referrals?

a. Please give the formal referral criteria for age range.

b. In practice what is the age of the oldest young adult on your caseload at the current time?

8. How many children and young people are currently on the active caseload of your hospice service (please exclude bereaved families)?

9. How many bereaved families are currently on the caseload of your hospice service?

Care needs

10a. Over the past year, how many children and/or young people have been provided with end of life care by your hospice service? (For the past year for which you collect this data). Please include all admissions/support for end of life care even for those children/young people whose condition improved.

10b. Over the past year, how many children and/or young people provided with end of life care died?

10c. Over the past year how many children and/or young people were supported during compassionate extubation by your hospice service.

Q11a. At the current time estimate the % of children on your hospice case load who have the following care needs.

11b. Can these care needs be supported in your hospice/by your hospice team in other settings, either within your own team of with the support of other professionals?

Care need	A. % Estimate of proportion of children on case load	B. Can this care need be supported by your hospice service<br>Yes/No
a. Intravenous medications		
b. TPN		
c. Any type of assisted ventilation (including Cpap and Bipap)		
d. Blood or platelet transfusion		
e. Peritoneal dialysis		
f. Full ventilation in the community (a child or young person discharged from hospital with full ventilation via tracheostomy).		

12. Are there any other forms of care needs or factors impacting on care for children and young people on your hospice caseload you would like to describe?

Overall model of medical service provision

13a. Which of the following broad categories describes the **overall model of your hospice service?** *Please indicate one which best applies*

a. Community hospice service (i.e. mainly supporting patients and families in their own homes).
b. Hospice with inpatient beds for children
c. Hospice with inpatient beds for children and young adults
d. Joint inpatient bed unit and community /outreach service for children.
e. Joint inpatient bed unit and community/ outreach service for children and young adults.
f. Children's hospice unit as part of an adult hospice

Defining children as up to 18 yrs of age and young adults as 18yrs – 25yrs.

13bi. How many inpatient hospice bed units does this interview represent?

13bii: How many community hospice services does this data represent?

13biii: Are any of these services currently being developed or recently built?

14. Which of the following best describes the **model of medical service provision** for your hospice?

14a **For Community Children's Hospice services (no inpatient beds):**

a. Nurse led community hospice service with no formal arrangement for medical service provision.
b. Nurse led community hospice service with formal arrangement with a General Practice surgery.
c. Nurse led community hospice service with formal arrangement with one or more GPs with special interest in PPM.
d. Nurse led community hospice service with Service Level Agreement (SLA) with another hospice for medical service provision.
e. Nurse led service with SLA with a hospital trust for medical service provision
f. Not applicable (hospice has inpatient bed)

14b **For children and young person's hospice service with inpatient beds unit**

a. Nurse led multidisciplinary team with formal arrangement with a General Practice surgery.
b. Nurse led multidisciplinary team with formal arrangement with one or more G.P with Special interest in PPM.
c. Consultant in PPM employed by hospice working with a team of doctors.
d. Consultant in PPM employed by hospital providing in reach into hospice as part of formal arrangement.
e. Team of doctors from a range of backgrounds providing daily sessions at the hospice (but no level 4 doctor integral to the team).
f. Not applicable (hospice has no inpatient beds)
g. Combination of above models (state which in free text description box)
h. None of above (describe below)

15. Please give a further description of the model of medical service provision for your hospice organisation:

Details of doctors working at the hospice

16. How many doctors are employed by your hospice?

17. How many doctors working at your hospice are employed by other organisations?

18. For doctors employed by organisations other than the hospice which organisation are they employed by?

Please state how many doctors working at the hospice are employed by the following organisations:

a. General practice

b. NHS: District general hospital

c. NHS: Tertiary children's hospital

d. Community Trust

19a. How many hours per week do doctors work at the hospice/ for the hospice service in work directly related to the care of children and families (either in the hospice, home, or hospital) excluding on call?
(*Multiply number of hours by number of doctors if more than one doctor working).*

19b. What pattern of work do your doctors have for their regular hours (not on call)?

a. A session per day (2 – 4 hours)
b. Full 9 – 5 days on weekdays
c. Full 9 – 5 days every weekday plus as required visits out of hours and at weekends.
d. Full 9 – 5 days every day
e. No regular hours: responsive to need

19c. Is there anything else you would like to explain about the way in which your doctors work?

20a. How many hours per week are doctors working for the hospice available for telephone advice to nursing and other multidisciplinary team members if needed?

20b What is the pattern of doctor's availability for telephone advice?

a. Weekdays 9 – 5
b. Everyday 9 – 5
c. 24 hours a day on weekdays, not at weekends
d. 24/7 (all the time)
e. No fixed pattern (as needed/ad hoc)

21a. How many hours per week are doctors working for the hospice available to see patients face-to-face as requested by nursing and other multidisciplinary team members?

21b What is the pattern of doctors' availability to see patients face to face on request.

a. Weekdays 9 – 5
b. Everyday 9 – 5
c. 24 hours a day on weekdays, not weekends
d. 24/7 (all the time)
e. No fixed pattern (ad hoc/as needed)

22. How many consultants in paediatric palliative medicine **work at the hospice?

*** Definition of consultant in paediatric palliative medicine given*

23. Who is or are s/he /they employed by for their hospice work? (state the number of consultants for each)

a. Hospice

b. Hospital

c. Joint post

d. Clinical commissioning group

24. What is the pattern of work for consultants in paediatric palliative medicine at the hospice? (Indicate all that apply)

a. Specified hospice sessions including face to face patient / family contact and medical review

b. Available for telephone advice

c. Available to visit if requested

d. Other (specify)

e. Not applicable (No consultant post)

Availability and type of 24/7 medical advice

25. Does the hospice service have access to medical advice 24/7?

Yes/ No

26.Which of the following best describes the arrangement for 24/7 medical advice at your hospice organisation?

a. 24/7 on call rota of doctors run by hospice
b. Service Level Agreement (SLA) with a G.P practice or cooperative
c. SLA with another children's hospice service
d. SLA with an adult hospice service
e. SLA with secondary children's hospital
f. SLA with tertiary children's hospital
g. No access to medical advice
h. 24/7 but one person therefore not sustainable 24/7

27. What type of advice does the on-call system cover? Please indicate all relevant options.

a. Telephone advice
b. Face to face patient / family review in hospice
c. Face to face patient / family review in hospital
d. Face to face patient / family review in community

28. What medical advice are the doctors providing 24/7 medical advice giving? Please indicate all relevant options.

a. Medical advice on general issues
b. General Paediatric Palliative medical advice
c. Specialist paediatric palliative medical advice

29. What is the training level in paediatric palliative medicine of the doctors giving advice? * For definition of levels

a. Levels 1 and 2
b. Level 3
c. Level 4
d. Both level 3 and 4 (a two-tier system of advice as needed)
e. Don't know
f. Not applicable (no access to medical advice)
g. Level 1, 2 and 3 (depending who is on call)

*Definition of levels **

30. If the hospice runs a 24/7 on-call rota which patients are covered? *Please indicate all that apply.*

a. Children/ young people at home who are already known to the hospice service
b. Children /young people at home not yet known to the hospice service
c. Children/ young people in inpatient bed hospice unit
d. Children/ young people in hospital who are already known to the hospice service
e. Children/young people in hospital who are not yet known to the hospice service

31. If the hospice runs a 24/7 on call rota who can normally contact medical staff for advice? Please indicate all relevant options

a. Nurses working for the hospice
b. All multidisciplinary team working for the hospice
c. Parents whose child is under the care of the hospice service
d. Parents whose child is not under the care of the hospice service
e. Professionals outside of hospice service

Training, experience and roles of doctors working for the hospice

32. How many of the doctors working for your hospice have developed the following level of competency?

a. Level three Paediatric Palliative Medicine *

b. Level four Paediatric Palliative Medicine *

33. Which medical specialties do doctors working at the hospice come from? (Indicate the core specialism for each doctor)
Please indicate the number of doctors for all that apply:

a. G.P
b. G.P with special interest in paediatric palliative medicine
c. Community paediatrics
d. Adult palliative medicine
e. Paediatric palliative medicine
f. Paediatric oncology
g. Paediatric intensive care
h. Paediatrics (of any specialty) with special interest in PPM
i. Other e.g., neurology, learning disability (please specify):

34. Do doctors who work for the hospice provide in reach into hospital(s)?

Yes/ No

35. How many of each of the following types of hospital do the doctors provide in reach for?

a. District general hospital
b. Tertiary children's hospital
c. Neonatal Unit

36. If the doctors working for the hospice provide in reach what are the arrangements for this?

a. Formal arrangement with honorary contract, funded
b. Formal arrangement with honorary contract, non-funded
c. Informal arrangement with no contract or funding
d. No in reach
e. Don't know
f. Employed by hospital in main post

37. Do doctors who work for the hospice provide outreach into the community?

Yes/ No

38. What are the arrangements for this out-reach?

a. Formal arrangement with honorary contract, funded
b. Formal arrangement with honorary contract, non funded
c. Informal arrangement with no contract or funding
d. No outreach
e. Don't know

39. Which of the following roles do doctors working at the hospice (in any capacity) undertake?

Please indicate all that apply.

a. Writing of medication charts
b. Prescribing new medications for patients
c. General medical review of patients (for issues not directly related to palliative care)
d. General PPM review of patients
e. Specialist PPM review of patients
f. Advice on symptom management
g. Writing and circulating symptom management plans
h. Writing discharge summaries following hospice admissions.
i. Discussion of advance care plans and end of life care plans with parent/ guardian and/or young person and /or the wider care team.
j. Arranging and providing medical input for admission to hospice for symptom control or end of life care.
k. Arranging and providing medical input for stepped discharge from hospital via hospice.
l. Arranging and providing medical input for emergency admissions to hospice (medical or social)
m. Medical review for family members (siblings/ parents)
n. Certification of death of patient
o. Contributing to ongoing extended team review of a patient.
p. Contributing to clinical governance and clinical policy development for the hospice.
q. Teaching and training at the hospice (both formal and informal)
r. Contributing to strategy and development in the wider medical community

Senior nursing posts

40a. Does your hospice have a nurse consultant or advanced nurse practitioner post?

Yes / No

40bi. Does your hospice have any non-medical prescribers?

Yes/No

40 bii: Number of non-medical prescribers
40c. If yes, what is their professional background:

a. Nursing
b. Pharmacist
c. Other (please specify)

41. If your hospice has an advanced nurse practitioner or consultant nurse what roles does s/he undertake?

Please Indicate all that apply:

a. Signing of medication charts
b. Prescribing new medications for patients
c. General health review of patients (for issues not directly related to palliative care)
d. General PPM review of patients (for issues relating to palliative care)
e. Specialist PPM review
f. Advice on symptom management
g. Writing symptom management plans
h. Discussion of advance care plans and emergency / end of life care plans with parent/ guardian or young person and/or the wider care team.
i. Arranging admission to hospice for symptom control or end of life care
j. Medical review for family members (siblings/ parents)
k. Certification of death of patient

42. Case scenarios:

Case scenario one: Response to need for symptom review during respite care

14-year-old Mark is under the care of your hospice service with a neurodegenerative condition. He experiences a deterioration in symptom control during a period of short break care (either at home or in your hospice). Mark is in distress, but it is unclear whether this is due to muscle spasm, seizures, or pain of another cause.

Overall: Describe how your hospice service would respond to this clinical case scenario.

Further questions:

1.1 Who would the respite carer call for advice in the first instance?

1.2 Would a doctor be contacted for advice?

1.3 If so who would this be and what is their level of PPM training?

1.4 If Mark needed review by a doctor how would this be arranged?

1.5 Mark needed a change in medication who would prescribe this?

Case scenario two: Response to need for rapid discharge from hospital to inpatient hospice for end of life care

Your hospice service receives a call from the local hospital's oncology team at 3pm on a Friday afternoon. The call is regarding Megan, an 8-year-old girl, with a diffuse pontine glioma. She has completed 1 year of treatment and is known to your hospice service. She has a VP shunt in situ, is in hospital and has headaches, can no longer swallow, and has a chest infection. The opinion of her lead oncologist is that she is in the last weeks of her life. Her family would like to be transferred to the hospice for end of life care, supported by your hospice service. It is a bank holiday weekend and they do not want to remain in hospital any longer.

Overall: Describe how your hospice service would respond to this clinical case scenario.

Further questions:

2.0 Overall how likely would it be that your hospice service could accept this referral and arrange transfer at this time:

a. Always
b. At a later stage with planning
c. May be able to accept but would depend on staffing
d. May be able to accept depending on medical cover
e. Never able to accept this type of request.

2.1 Who would take this initial call?

2.2 How would a decision be made about whether the hospice service can facilitate her rapid discharge from hospital?

2.3 Would a hospice doctor normally be involved in this decision?

2.4a If so, who?

2.4b And what is their level of PPM training?

2.5 Would a doctor or nurse from the hospice service be available and likely to visit? Megan and her family in hospital prior to discharge?

2.6 On arrival at the hospice who would be available to assess Megan?

2.7 Who would be most likely to lead on discussions about a symptom control plan with the family and document this plan?

2.8 Who might discuss and document an advance care plan / end of life care plan?

2.9 Who would prescribe medications?

At 3am on Sunday morning Megan's symptoms deteriorate with increased headaches and development of muscle spasms.

2.11 Who would be called for advice in the first instance?

2.12 Would a doctor be available to give telephone advice?

2.13 If so who would this be and what is their level of PPM training?

2.14 Would a doctor be available to visit and assess Megan?

Case scenario three: Response to need for rapid discharge from hospital to home for end of life care

Your hospice service receives a call from the local hospital's oncology team at 3pm on a Friday afternoon regarding Megan, an 8 year old girl, with a diffuse pontine glioma. She has completed 1 yr of treatment and is known to your hospice service. She has a VP shunt in situ, is in hospital, has headaches, can no longer swallow and has a chest infection. The opinion of her lead oncologist is that she is in the last weeks of her life. Her family would like to be discharged home for end of life care supported by your hospice service. It is a Bank Holiday weekend and they do not want to remain in hospital any longer.

Overall: Describe how your hospice service would respond to this clinical case scenario.

Further questions:

3.0 Overall how likely would it be that your hospice service could accept this referral and arrange transfer at this time:

a. Always

b. At a later stage with planning

c. May be able to accept but would depend on staffing

d. May be able to accept depending on medical cover

e. Never able to accept this type of request.

3.1 Who would take this initial call?

3.2 How would a decision be made about whether the hospice service can facilitate her rapid discharge from hospital?

3.3 Would a hospice doctor normally be involved in this decision

3.4a If so, who?

3.4b And what is their level of PPM training?

3.4 Would a doctor or nurse from the hospice service be available and likely to visit Megan and her family in hospital prior to discharge?

3.5 On arrival at home who would be available to assess Megan?

3.7 Who would be most likely to lead on discussions about a symptom control plan with the family and document this plan?

3.8 Who might discuss and document an advance care plan / end of life care plan for Megan?

3.9 Who would prescribe medications?

3.10 Who would ensure that a supply of emergency medicine is available in the home?

At 3am on Sunday morning Megan's symptoms deteriorate with increased headaches and development of muscle spasms.

3.11Who would parents call for advice in the first instance?

3.12Would a hospice doctor be available to give telephone advice?

3.13If so who would this be and what is their level of PPM training?

3.14Would a hospice doctor be available to visit and assess Megan if required?

Case scenario four: Response to requests for advice from a hospital consultant

A hospital paediatric consultant contacts the hospice on a weekday for advice regarding a patient, James, who is currently in hospital and is known to your hospice service. James is 12 years old and has a rare neurodegenerative condition with intermittent and progressive gut failure. He was admitted to hospital 2 days ago with abdominal pain and erratic absorption. The consultant is asking for advice on managing his pain and support in reviewing overall goals of care.

Overall: Describe how your hospice service would respond to this clinical case scenario.

Further questions:

4.1Who would respond to this request for advice?

4.2How would the request be responded to?

4.3 Would a member of the hospice team be available to visit James and his family in hospital?

4.4aIf yes, who is this likely to be?

4.4b And what form would the visit take?

4.5How would the hospice team respond to this request on a weekend?

*Definitions of levels of paediatric palliative medicine education and training

Full description of Levels of competency in paediatric palliative medicine from the Combined Curriculum (APPM Education Subgroup and RCPCH 2015)

For paediatricians, these would correspond to:
• Level 1: a doctor just completing a medical degree,
• Level 2: a paediatric specialist registrar who has completed core paediatric training
• Level 3: a consultant paediatrician who has gone on to general or subspecialty training in a related field and developed a special interest in paediatric palliative medicine
• Level 4: a consultant in specialist paediatric palliative medicine.

For children's hospice doctors, these would correspond to:
• Level 1: a doctor just completing a medical degree,
• Level 2: a children's hospice doctor after one year of experience,
• Level 3: a children's hospice medical director, or other established children's hospice doctor who has gained the Cardiff Diploma in paediatric palliative medicine or similar validated qualification ,
• Level 4: a few hospice medical directors (mainly historical leaders in subspecialty formation and development, with roles beyond the local hospice). Likely to have been awarded FRCPCH or similar distinction.

While the specific standards for each competency would, of course, depend on its nature, the following general principles were used to define the different levels:

- Level 1. Understand the basic principles, of paediatric palliative care
- Level 2. Apply basic principles of palliative medicine to the care of children specifically. Recognise reversible causes of symptoms in children, whether with a life-limiting condition or not.
- Level 3. Be able to manage most common symptoms safely and effectively. Be prepared to recognise need for specialist help and access it where necessary.
- Level 4. Manage uncommon symptoms; understand principles in order to develop a logical approach even where there is no evidence base. Considerable emphasis on leading and developing services within and beyond the local hospice, and on supporting and teaching other professionals involved with children with life-limiting conditions who are not trained in palliative medicine. This level will probably only be seen to be achieved if the doctor has obtained FRCPCH or similar distinction
